# Eosinophil IL-5Rα/JAK2/STAT5 Signaling Contributes to Epithelial–Mesenchymal Transition in Eosinophilic Chronic Rhinosinusitis with Nasal Polyps

**DOI:** 10.3390/medicina62071360

**Published:** 2026-07-15

**Authors:** Hosung Choi, Hyunsu Choi, Jeong-Min Oh, Hyun Seok Lee, Soo Whan Kim, Byung Guk Kim, Dong Chang Lee

**Affiliations:** 1 Department of Otorhinolaryngology Head and Neck Surgery, College of Medicine, The Catholic University of Korea, Seoul 06591, Republic of Korea; thehosung@naver.com (H.C.); hsleesu@naver.com (H.S.L.); kshent@catholic.ac.kr (S.W.K.); coolkim@catholic.ac.kr (B.G.K.); 2 Clinical Research Institute, Daejeon St. Mary’s Hospital, Daejeon 34943, Republic of Korea; lolo@cmcnu.or.kr (H.C.); yejmdh@cmcnu.or.kr (J.-M.O.)

**Keywords:** rhinosinusitis, eosinophils, nasal polyps, interleukin-5, epithelial–mesenchymal transition

## Abstract

*Background and Objectives*: Eosinophilic chronic rhinosinusitis with nasal polyps (ECRSwNP) is characterized by type 2 inflammation, marked eosinophil infiltration, and enhanced epithelial–mesenchymal transition (EMT). Although interleukin-5 (IL-5) is central to eosinophil differentiation and activation, its role in EMT in human nasal epithelial cells (HNECs) remains unclear. This study aimed to elucidate the contribution of IL-5Rα/Janus kinase 2 (JAK2)/signal transducer and activator of transcription 5 (STAT5) signaling in eosinophils in EMT in ECRSwNP. *Materials and Methods*: Nasal mucosal tissues from control and ECRSwNP or non-ECRSwNP group patients (*n* = 12 each) were analyzed for type 2 cytokine, EMT marker, and IL-5Rα/JAK2/STAT5 axis component levels using Western blotting, immunohistochemistry, and quantitative real-time polymerase chain reaction. HL-60 cells were differentiated into eosinophil-like cells using butyric acid and stimulated with IL-5, and HNECs were co-cultured with undifferentiated, differentiated, or IL-5-activated differentiated HL-60 cells. EMT induction and migration were assessed using immunofluorescence, wound-healing assays, and Western blotting. IL-5RA, JAK2, or STAT5 was silenced using small interfering RNA to determine pathway dependency. *Results*: ECRSwNP tissues showed elevated type 2 cytokine and EMT marker expression and enhanced IL-5Rα/JAK2/STAT5 pathway activation. Co-culture with IL-5-activated differentiated HL-60 cells induced EMT in HNECs, evidenced by decreased E-cadherin and zonula occludens-1, increased N-cadherin and vimentin levels, and enhanced migration. Moreover, silencing IL-5RA, JAK2, or STAT5 significantly attenuated these effects. *Conclusions*: IL-5-activated IL-5Rα/JAK2/STAT5 signaling in eosinophils may contribute to EMT in HNECs. Thus, this pathway could be a potential therapeutic target for tissue remodeling and polyp formation in type 2 chronic rhinosinusitis.

## 1. Introduction

Chronic rhinosinusitis (CRS) is a chronic inflammatory condition of the sinonasal passages that persists for 12 weeks or more [1]. Recently, CRS classification has shifted from the conventional categorization based on nasal polyp presence to an endotype-driven approach that distinguishes between non-type 2 and type 2 inflammatory patterns [2,3], driven by the need to better understand disease heterogeneity and improve the ability to predict recurrence risk and treatment response. From an immunological nomenclature standpoint, type 2 chronic rhinosinusitis with nasal polyps (CRSwNP) may also be understood within the framework of a type IVb hypersensitivity-associated type 2 immune response, in which T helper 2 cells, group 2 innate lymphoid cells, eosinophils, and type 2 cytokines, including interleukin-4 (IL-4), interleukin-5 (IL-5), and interleukin-13 (IL-13), collectively contribute to chronic eosinophilic inflammation [4]. Eosinophilic chronic rhinosinusitis with nasal polyps (ECRSwNP), a representative type 2 endotype, typically exhibits eosinophilic infiltration of sinonasal tissues along with a predominant type 2 inflammatory profile, rendering it particularly refractory to conventional treatment approaches, including surgery [5,6].

Epithelial–mesenchymal transition (EMT) is a biological process whereby epithelial cells lose their characteristics and develop mesenchymal features, resulting in increased invasiveness, migratory capacity, and extracellular matrix (ECM) component production [7]. Type 2 EMT, one of the three classifications of EMT, is predominantly initiated by inflammation-mediated tissue injury. It plays a role in both fibrosis and tissue regeneration and is implicated in tissue remodeling associated with chronic airway diseases, including CRS, asthma, and chronic obstructive pulmonary disease [7,8].

Recent studies have suggested that EMT plays a role in nasal polyp formation and may represent a promising therapeutic target [9,10,11]. The degree of eosinophil infiltration has been demonstrated to be closely related to the extent of EMT, indicating the importance of eosinophils in EMT. Moreover, the degree of EMT is higher in CRSwNP than in chronic rhinosinusitis without nasal polyps, with the highest levels observed in ECRSwNP [12].

IL-5 is a key cytokine that plays a crucial role in the activation, survival, and differentiation of eosinophils. When IL-5 binds to the IL-5 receptor α (IL-5Rα) chain on the eosinophil surface, it forms a receptor complex with the common β chain. This binding event sets off a cascade of intracellular protein phosphorylation reactions, ultimately leading to changes in gene transcription [13,14,15]. A well-characterized pathway in this process involves IL-5 binding to IL-5Rα to activate Janus kinase 2 (JAK2) and subsequently signal transducer and activator of transcription 5 (STAT5) in a signaling cascade that is essential for downstream signal transduction [14,15].

However, despite the established role of IL-5 in eosinophil biology, studies investigating its involvement in EMT in ECRSwNP remain limited. Therefore, this study aimed to investigate whether IL-5 induces EMT in ECRSwNP, with particular emphasis on the role of the eosinophilic IL-5Rα/JAK2/STAT5 signaling pathway.

## 2. Materials and Methods

### 2.1. Study Subjects and Ethical Approval

We conducted ex vivo analyses of sinonasal tissue samples from patients aged ≥ 19 years who underwent endoscopic sinonasal surgery, including functional endoscopic sinus surgery, septoplasty, or inferior turbinoplasty, at our institution between September 2024 and December 2025. The exclusion criteria were primary ciliary dyskinesia, cystic fibrosis, immunodeficiency, systemic corticosteroid use within 4 weeks, or neoplastic lesions. After written informed consent was obtained in accordance with the Declaration of Helsinki, discarded surgical specimens (nasal polyps, uncinate processes, and inferior turbinate tissue) were collected for analysis.

CRSwNP was diagnosed according to the European Position Paper on Rhinosinusitis and Nasal Polyps (EPOS) 2020 criteria, incorporating clinical symptoms, nasal endoscopic findings, and radiological assessment by computed tomography.

To distinguish ECRSwNP from non-ECRSwNP, tissue eosinophil counts were determined in hematoxylin and eosin (H&E)-stained sections. ECRSwNP was diagnosed if there were ≥70 eosinophils per high-power field (HPF, 400× magnification), with counts averaged from at least five sections per specimen. This cutoff was selected based on the widely used Japanese Epidemiological Survey of Refractory Eosinophilic Chronic Rhino-sinusitis (JESREC) criteria, which were developed in an East Asian population, although different tissue eosinophil thresholds have been proposed in other geographic settings [16,17,18]. Ethical approval was obtained from the Institutional Review Board (IRB policy No. DC24TISI0055, 3 September 2024).

### 2.2. H&E Staining and Eosinophil Quantification

Nasal mucosal specimens were fixed in 10% neutral-buffered formalin, paraffin-embedded, and sectioned at 4 µm using a rotary microtome. After deparaffinization with xylene and rehydration in graded ethanol, sections were stained with H&E for the assessment of epithelial architecture, tissue morphology, and inflammatory cell infiltration. For quantification, eosinophils were counted in five randomly selected, non-overlapping HPFs (400× magnification) per specimen and expressed as the mean number per HPF.

### 2.3. Immunohistochemistry (IHC) and Quantitative Analysis

Formalin-fixed, paraffin-embedded nasal mucosal tissues were sectioned at 4 µm, deparaffinized, and subjected to antigen retrieval in citrate buffer (pH 6.0) using microwave heating. Endogenous peroxidase activity was blocked with 3% hydrogen peroxide, followed by incubation with 5% bovine serum albumin. The sections were incubated overnight at 4 °C with primary antibodies against IL-5Rα (Abcam, Cambridge, UK), Ki67 (Abcam), E-cadherin (Abcam), or vimentin (Cell Signaling Technology, Danvers, MA, USA), followed by biotin-conjugated secondary antibody for 1 h. Immunoreactivity was visualized using the Vectastain ABC kit and ImmPACT Vector Red chromogen (Vector Laboratories, Burlingame, CA, USA), and nuclei were counterstained with hematoxylin. Images were obtained at 400× magnification and analyzed using ImageJ software (version 1.54p; National Institutes of Health, Bethesda, MD, USA). For each marker, five randomly selected, non-overlapping HPFs were analyzed, and the proportion of immunopositive cells relative to total nucleated cells was calculated. Slides were evaluated independently by two observers blinded to clinical group allocation, and mean values were used for statistical analysis.

### 2.4. Quantitative Real-Time Polymerase Chain Reaction (qRT-PCR)

Total RNA was extracted from nasal mucosal tissues and HL-60 cells using the Easy-BLUE™ Extraction Kit (iNtRON Biotechnology, Seongnam, Republic of Korea). RNA concentration and purity were measured spectrophotometrically, and complementary DNA was synthesized using 1 µg of RNA. qRT-PCR was performed using SYBR Green chemistry with gene-specific primers. mRNA expression of IL-4, IL-5, IL-13, interferon gamma (IFN-γ), interleukin-17 (IL-17), interleukin-22 (IL-22), and IL-5RA was analyzed in nasal tissues, whereas IL-5RA and C-C chemokine receptor type 3 (CCR3) were evaluated in HL-60 cells. Glyceraldehyde-3-phosphate dehydrogenase (GAPDH) served as the internal reference gene, and relative expression was calculated using the 2^−ΔΔCt^ method. Primer sequences are provided in Supplementary Table S1.

### 2.5. Western Blotting

Protein extracts from nasal tissues and HL-60 cells were prepared using lysis buffer containing protease and phosphatase inhibitors. Equal amounts of protein (20 µg) were separated using sodium dodecyl sulfate–polyacrylamide gel electrophoresis (SDS-PAGE) and transferred to nitrocellulose membranes. After blocking, membranes were incubated overnight at 4 °C with primary antibodies against IL-5Rα, phosphorylated-JAK 2 (p-JAK2), JAK2, phosphorylated-STAT5 (p-STAT5), STAT5, E-cadherin, zonula occludens-1 (ZO-1), N-cadherin, vimentin, and GAPDH, followed by horseradish peroxidase–conjugated secondary antibodies. Bands were visualized using enhanced chemiluminescence and quantified using ImageJ. Phosphorylated protein levels were normalized to the corresponding total protein forms (p-JAK2/JAK2 and p-STAT5/STAT5), and total protein levels were normalized to GAPDH.

### 2.6. HL-60 Cell Culture, Differentiation, and IL-5 Stimulation

Clone 15 HL-60 cells, a subline of the human promyelocytic leukemia cell line HL-60, were purchased from the American Type Culture Collection (ATCC, Manassas, VA, USA; Cat. No. CRL-1964) and cultured in RPMI-1640 medium (Gibco, Grand Island, NY, USA) supplemented with 10% fetal bovine serum and 1% penicillin-streptomycin. Eosinophil-like differentiation was induced using 0.5 mM of sodium butyrate (Sigma-Aldrich, St. Louis, MO, USA) for 7 days, with medium changes performed every 48 h. Differentiation was confirmed by increased IL-5RA and CCR3 mRNA expression. Differentiated cells were stimulated with recombinant human IL-5 (10 ng/mL; PeproTech, Rocky Hill, NJ, USA) for 24 h, while undifferentiated cells maintained in the same medium served as controls.

### 2.7. Small Interfering RNA (siRNA) Transfection of Differentiated HL-60 Cells

Differentiated HL-60 cells were transfected with siRNAs targeting IL-5RA, JAK2, or STAT5, with scrambled siRNA (si-Control) as control. Cells (1 × 10^6^ cells/mL) were transfected with 50 nM of siRNA using Santa Cruz siRNA Transfection Reagent according to the manufacturer’s protocol. After 6 h, the medium was replaced, and cells were allowed to recover for 24 h before stimulation with recombinant human IL-5 (10 ng/mL) for 24 h. Cells and supernatants were collected for Western blotting analysis of IL-5Rα, p-JAK2, JAK2, p-STAT5, and STAT5 and for enzyme-linked immunosorbent assay (ELISA) measurement of eosinophil cationic protein (ECP), eosinophil peroxidase (EPO), IL-13, and transforming growth factor beta 1 (TGF-β1).

### 2.8. Human Nasal Epithelial Cell (HNEC) Culture and Co-Culture with HL-60 Cells

Primary HNECs were obtained from PromoCell GmbH (Heidelberg, Germany; Cat. No. C-12620; Lot No. 477Z049). According to the donor information provided by the manufacturer, the cells were isolated from normal human nasal mucosa obtained from a 78-year-old Caucasian female donor and were characterized as cytokeratin-positive epithelial cells. HNECs were maintained in an Airway Epithelial Cell Growth Medium Kit (PromoCell; Cat. No. C-21160), consisting of Airway Epithelial Cell Basal Medium supplemented with the manufacturer-provided SupplementPack. Cells at passages 3–5 were used for all experiments. Cells were seeded in 6-well plates (2 × 10^5^ cells/well) and grown to 80–90% confluence before co-culture. For paracrine studies, HL-60 cells (undifferentiated, butyrate-differentiated, or IL-5–stimulated differentiated) were placed in Transwell inserts with 0.4 µm pore membranes (Corning Inc., Corning, NY, USA) at a 1:2 ratio (HNECs:HL-60), allowing soluble factor exchange without direct contact. Similarly, to assess IL-5Rα/JAK2/STAT5 signaling, IL-5-activated differentiated HL-60 cells transfected with si-Control, si-IL-5RA, si-JAK2, or si-STAT5 were added. After 48 h, HNECs were harvested for immunofluorescence, Western blotting analysis of EMT markers, and wound-healing assays.

### 2.9. Immunofluorescence Staining and Quantitative Analysis

After 48 h of co-culture, HNECs were fixed with 4% paraformaldehyde, permeabilized, and blocked before incubation with primary antibodies against E-cadherin and vimentin. The cells were then incubated with fluorophore-conjugated secondary antibodies (Invitrogen, Carlsbad, CA, USA) and counterstained with 4′,6-diamidino-2-phenylindole (DAPI). Images were obtained at 400× magnification using identical exposure settings. Mean fluorescence intensity was quantified using ImageJ from 5 to 7 randomly selected fields per condition after background correction and normalized to the respective control group.

### 2.10. ELISA

Following the various experimental treatments (undifferentiated, butyrate-differentiated, IL-5–stimulated differentiated, or siRNA-transfected differentiated cells with IL-5), HL-60 cell suspensions were centrifuged (1000× *g*, 10 min, 4 °C), and supernatants were collected, clarified, and stored at −80 °C. Levels of ECP, EPO, IL-13, and TGF-β1 were quantified using commercial ELISA kits (R&D Systems, Minneapolis, MN, USA). Absorbance was measured at 450 nm with 570 nm correction using a microplate reader (Bio-Rad Laboratories, Hercules, CA, USA). Concentrations were calculated using standard curves (pg/mL) and normalized to viable cell count values.

### 2.11. Wound-Healing Assay

Cells were grown to confluence in a Culture-Insert 2 Well in µ-Dish (ibidi GmbH, Gräfelfing, Germany); thereafter, the insert was removed with sterile forceps to create a uniform gap, and detached cells were washed away using phosphate-buffered saline. Serum-free medium was used to minimize proliferation. For paracrine co-culture, HL-60 cells under different conditions (undifferentiated, butyrate-differentiated, IL-5-activated differentiated, or siRNA-transfected IL-5-activated differentiated) were placed in 0.4 µm Transwell inserts above wounded HNECs. Images were captured at 0, 24, and 48 h using an inverted microscope. Gap closure was quantified with ImageJ by measuring the distance between wound edges and expressed as percentage reduction relative to 0 h.

### 2.12. Statistical Analysis

Each experiment was performed with a minimum of three independent biological replicates. Results are expressed as mean ± standard error of the mean (SEM) values. Intergroup differences were assessed using a one-way analysis of variance (ANOVA) with Tukey’s post hoc test for multiple comparisons. All statistical evaluations were performed using GraphPad Prism, version 5.03 (GraphPad Software, La Jolla, CA, USA). Statistical significance was defined as *p* < 0.05.

## 3. Results

### 3.1. Enhanced Eosinophilic Infiltration and Type 2 Inflammatory Profile in ECRSwNP

**Figure 1:**
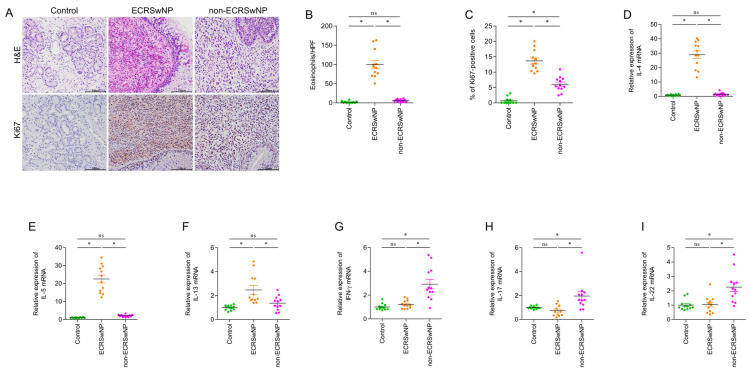
**Histological and transcriptional features in ECRSwNP and non-ECRSwNP.** (**A**) Representative histological images of nasal mucosal tissues. The upper row shows H&E staining for morphological assessment and inflammatory cell infiltration, and the lower row shows Ki67 IHC for epithelial proliferation (red chromogen). Magnification: 400×; scale bar = 100 µm. (**B**) Quantification of eosinophil infiltration expressed as the number of eosinophils per HPF in H&E-stained sections in panel (**A**). (**C**) Quantification of epithelial proliferation expressed as the percentage of Ki67-positive epithelial cells from the IHC staining in panel (**A**). (**D**–**F**) Relative mRNA expression of type 2 cytokines IL-4 (**D**), IL-5 (**E**), and IL-13 (**F**) determined using qRT-PCR and normalized to GAPDH. (**G**–**I**) Relative mRNA expression of type 1 and type 3 cytokines IFN-γ (**G**), IL-17 (**H**), and IL-22 (**I**) determined by qRT-PCR and normalized to GAPDH. In all scatter plots (**B**–**I**), each dot represents an individual subject (*n* = 12 per group); horizontal bars indicate the mean ± SEM. Statistical analysis was performed using one-way ANOVA followed by Tukey’s post hoc test. * *p* < 0.05 was considered statistically significant; ns indicates statistically not significant. Abbreviations: ANOVA, analysis of variance; ECRSwNP, eosinophilic chronic rhinosinusitis with nasal polyps; GAPDH, glyceraldehyde-3-phosphate dehydrogenase; H&E, hematoxylin and eosin; HPF, high-power field; IFN-γ, interferon-γ; IHC, immunohistochemistry; IL, interleukin; qRT-PCR, quantitative real-time polymerase chain reaction; SEM, standard error of the mean.

Patients were classified into control (*n* = 12), non-ECRSwNP (*n* = 12), and ECRSwNP (*n* = 12) groups, and their baseline demographic and clinical characteristics are summarized in Supplementary Table S2. Tissue samples from these groups were then analyzed for inflammatory infiltration, epithelial proliferation, and cytokine profiles. H&E staining revealed markedly increased eosinophil infiltration in the ECRSwNP group (99.7 ± 10.5 cells/HPF) compared with the non-ECRSwNP and control groups (each *p* < 0.001), with no difference between the latter two groups (*p* = 0.89) (Figure 1A,B). Ki-67 staining showed the highest proliferative activity in the ECRSwNP group and intermediate and minimal levels in the non-ECRSwNP and control groups, respectively (Figure 1A–C).

Type 2 cytokine (IL-4, IL-5, and IL-13) levels were markedly elevated in ECRSwNP (Figure 1D–F), whereas non-ECRSwNP tissues exhibited higher expression of IL-17, IL-22, and IFN-γ (Figure 1G–I), indicating distinct inflammatory endotypes.

### 3.2. Elevated EMT Markers and IL-5Rα/JAK2/STAT5 Pathway Activation in ECRSwNP Tissues

**Figure 2:**
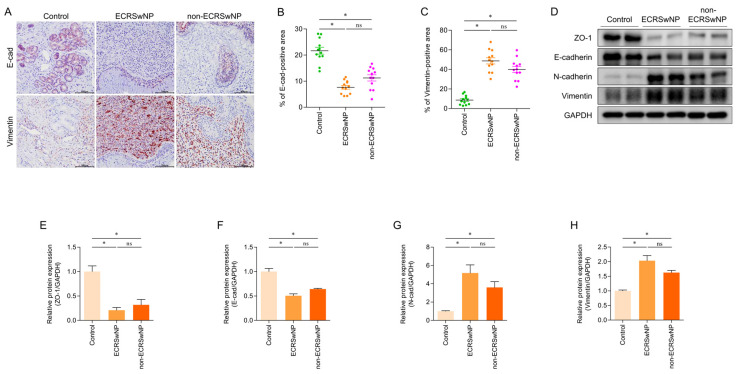
**Elevated expression of EMT markers in ECRSwNP and non-ECRSwNP groups.** (**A**) Representative IHC images of epithelial and mesenchymal markers in nasal mucosal tissues. The upper row shows staining for the epithelial marker E-cadherin, and the lower row shows staining for the mesenchymal marker vimentin (red chromogen). Magnification: 400×; scale bar = 100 µm. (**B**,**C**) Quantitative analysis of EMT markers from the IHC staining in panel (**A**), shown as the percentage of positive area for E-cadherin (**B**) and vimentin (**C**). (**D**) Representative Western blotting images showing epithelial markers (ZO-1 and E-cadherin) and mesenchymal markers (N-cadherin and vimentin) in nasal mucosal tissue lysates from the control, ECRSwNP, and non-ECRSwNP groups. GAPDH served as the loading control; two representative samples are shown per group. (**E**–**H**) Densitometric quantification of ZO-1 (**E**), E-cadherin (**F**), N-cadherin (**G**), and vimentin (**H**) protein expression normalized to GAPDH. In scatter plots (**B**,**C**), each dot represents an individual subject (*n* = 12 per group). For Western blot quantification shown in the bar graphs (**E**–**H**), densitometric analyses were performed using all tissue specimens from each group (*n* = 12 per group), although only two representative samples per group are shown in panel (**D**); bars indicate the mean ± SEM. Statistical analysis was performed using a one-way ANOVA followed by Tukey’s post hoc test. * *p* < 0.05 was considered statistically significant for pairwise comparisons; ns indicates statistically not significant. Abbreviations: ANOVA, analysis of variance; E-cad, E-cadherin; ECRSwNP, eosinophilic chronic rhinosinusitis with nasal polyps; EMT, epithelial–mesenchymal transition; GAPDH, glyceraldehyde-3-phosphate dehydrogenase; IHC, immunohistochemistry; N-cad, N-cadherin; SEM, standard error of the mean; ZO-1, zonula occludens-1.

IHC and Western blot analyses of representative marker levels were used to assess EMT across groups. IHC showed decreased E-cadherin expression in the ECRSwNP (7.7 ± 0.7%) and non-ECRSwNP groups (11.2 ± 1.2%) compared with that in the control group (21.7 ± 1.3%; each *p* < 0.001), whereas vimentin expression was increased in the ECRSwNP (48.7 ± 3.2%) and non-ECRSwNP groups (40.0 ± 3.1%) relative to that in the control group (8.7 ± 1.4%; each *p* < 0.001). No significant differences were observed between the two disease groups (both *p* = 0.07) (Figure 2A–C).

Western blotting analysis yielded similar results. ZO-1 expression decreased to 0.21 ± 0.06-fold in the ECRSwNP group and 0.32 ± 0.11-fold in the non-ECRSwNP group (each *p* < 0.001), while E-cadherin expression was reduced to 0.51 ± 0.04-fold (*p* < 0.001) and 0.64 ± 0.02-fold (*p* = 0.002) in the ECRSwNP and non-ECRSwNP groups, respectively, compared with the controls. Conversely, N-cadherin expression increased to 5.17 ± 0.89-fold (*p* < 0.001) and 3.60 ± 0.63-fold (*p* = 0.03), while vimentin expression increased to 2.03 ± 0.18-fold (*p* < 0.001) and 1.63 ± 0.07-fold (*p* = 0.003) in the ECRSwNP and non-ECRSwNP groups, respectively, compared with the controls. No significant intergroup differences were detected (all *p* > 0.05) (Figure 2D–H).

**Figure 3:**
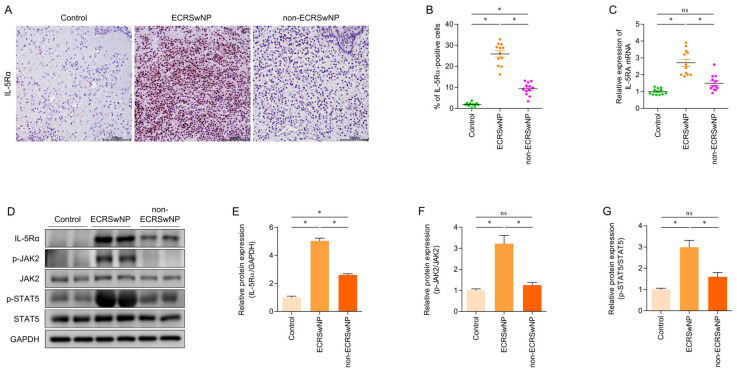
**Upregulation of IL-5Rα and activation of the JAK2/STAT5 signaling pathway in ECRSwNP.** (**A**) Representative IHC images showing IL-5Rα expression in nasal mucosal tissues from control subjects and patients with ECRSwNP or non-ECRSwNP (red chromogen). Magnification: 400×; scale bar = 100 µm. (**B**) Quantitative analysis of IL-5Rα expression from the IHC staining in panel (**A**), presented as the percentage of IL-5Rα-positive cells. (**C**) Relative IL-5RA mRNA expression in nasal mucosal tissues determined using qRT-PCR and normalized to GAPDH. (**D**) Representative Western blotting images showing IL-5Rα protein expression and phosphorylation of downstream signaling molecules (p-JAK2/JAK2 and p-STAT5/STAT5) in nasal mucosal tissue lysates. GAPDH served as the loading control; two representative samples are shown per group. (**E**–**G**) Densitometric quantification of IL-5Rα protein expression normalized to GAPDH (**E**) and phosphorylation levels of JAK2 (**F**) and STAT5 (**G**) expressed as the ratio of phosphorylated to total protein. In scatter plots (**B**,**C**), each dot represents an individual subject (*n* = 12 per group). For Western blot quantification shown in the bar graphs (**E**–**G**), densitometric analyses were performed using all tissue specimens from each group (*n* = 12 per group), although only two representative samples per group are shown in panel (**D**); bars indicate the mean ± SEM. Statistical analysis was performed using a one-way ANOVA followed by Tukey’s post hoc test. * *p* < 0.05 was considered statistically significant for pairwise comparisons; ns indicates statistically not significant. Abbreviations: ANOVA, analysis of variance; ECRSwNP, eosinophilic chronic rhinosinusitis with nasal polyps; GAPDH, glyceraldehyde-3-phosphate dehydrogenase; IHC, immunohistochemistry; IL-5Rα, interleukin-5 receptor α; JAK2, Janus kinase 2; p-JAK2, phosphorylated Janus kinase 2; p-STAT5, phosphorylated signal transducer and activator of transcription 5; qRT-PCR, quantitative real-time polymerase chain reaction; SEM, standard error of the mean; STAT5, signal transducer and activator of transcription 5.

IHC and mRNA analysis showed that IL-5Rα expression was significantly elevated in the ECRSwNP group (Figure 3A–C). Western blotting further demonstrated increased IL-5Rα expression and higher p-JAK2/JAK2 and p-STAT5/STAT5 ratios in the ECRSwNP group compared with the non-ECRSwNP group and controls (Figure 3D–G), indicating activation of the IL-5Rα/JAK2/STAT5 pathway.

### 3.3. IL-5-Activated Differentiated HL-60 Cells Promote EMT and Cell Migration in HNECs via Paracrine Interaction

To determine whether butyric acid induces eosinophil-like differentiation of HL-60 cells and whether IL-5 further activates these cells, we evaluated eosinophil-specific markers, secreted mediators, and IL-5Rα/JAK2/STAT5 signaling. Butyric acid treatment increased the expression of eosinophil markers, including IL-5RA and CCR3, while IL-5 stimulation of differentiated HL-60 cells enhanced mediator secretion and activation of the IL-5Rα/JAK2/STAT5 pathway (Supplementary Figure S1).

**Figure 4:**
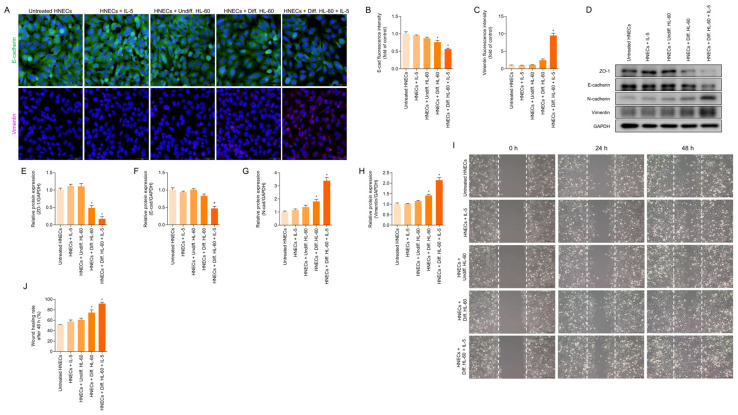
**IL-5-activated differentiated HL-60 cells induce EMT and enhance migration in HNECs via paracrine interaction.** (**A**) Representative immunofluorescence images showing epithelial (E-cadherin) and mesenchymal (vimentin) markers in HNECs under the indicated conditions. E-cadherin is shown in green (upper row), vimentin in red (lower row), and nuclei are counterstained with DAPI (blue). Magnification: 400×; scale bar = 100 µm. (**B**,**C**) Quantitative analysis of fluorescence intensity from the immunofluorescence staining in panel (**A**). Expression is presented as fold change relative to control (untreated HNECs) for E-cadherin (**B**) and vimentin (**C**). (**D**) Representative Western blotting images showing epithelial markers (ZO-1, E-cadherin) and mesenchymal markers (N-cadherin and vimentin) in HNECs harvested from co-culture experiments. GAPDH served as the loading control. (**E**–**H**) Densitometric quantification of ZO-1 (**E**), E-cadherin (**F**), N-cadherin (**G**), and vimentin (**H**) protein expression normalized to GAPDH. (**I**) Representative wound-healing assay images of HNECs under various conditions at 0, 24, and 48 h. (**J**) Quantitative analysis of HNEC migratory capacity expressed as the wound-healing rate (%) at 48 h, calculated from the images in panel (**I**). Data in bar graphs (**B**,**C**,**E**–**H**,**J**) are presented as the mean ± SEM from three independent biological replicates, each performed as a separate co-culture experiment. Statistical analysis was performed using a one-way ANOVA followed by Tukey’s post hoc test. * *p* < 0.05 was considered statistically significant compared with the control (untreated HNECs). Abbreviations: ANOVA, analysis of variance; DAPI, 4′,6-diamidino-2-phenylindole; Diff. HL-60, butyric acid-differentiated HL-60 cells; E-cad, E-cadherin; EMT, epithelial–mesenchymal transition; GAPDH, glyceraldehyde-3-phosphate dehydrogenase; HNECs, human nasal epithelial cells; IL-5, interleukin-5; N-cad, N-cadherin; SEM, standard error of the mean; Undiff. HL-60, undifferentiated HL-60 cells; ZO-1, zonula occludens-1.

To assess paracrine effects on EMT in HNECs, we used a non-contact Transwell co-culture system comparing untreated HNECs with HNECs co-cultured with IL-5 alone, undifferentiated HL-60 cells, differentiated HL-60 cells, or differentiated HL-60 + IL-5. E-cadherin fluorescence intensity was significantly reduced in both the HNECs + differentiated HL-60 group (0.76 ± 0.05-fold, *p* = 0.009) and the HNECs + differentiated HL-60 + IL-5 group (0.56 ± 0.02-fold, *p* < 0.001) compared with the controls. In contrast, vimentin fluorescence intensity was significantly increased only in the IL-5-treated group (9.51 ± 0.65-fold, *p* < 0.001) (Figure 4A–C).

Western blotting analysis confirmed these results. ZO-1 expression was reduced in both the HNECs + differentiated HL-60 group (0.49 ± 0.06-fold, *p* = 0.002) and the HNECs + differentiated HL-60 + IL-5 group (0.17 ± 0.06-fold, *p* < 0.001). E-cadherin expression decreased only in the HNECs + differentiated HL-60 + IL-5 group (0.47 ± 0.06-fold, *p* < 0.001). Conversely, N-cadherin expression increased to 1.81 ± 0.16-fold (*p* = 0.01) in the HNECs + differentiated HL-60 group and to 3.39 ± 0.23-fold (*p* < 0.001) in the HNECs + differentiated HL-60 + IL-5 group. Similarly, vimentin expression rose to 1.42 ± 0.06-fold (*p* = 0.01) in the HNECs + differentiated HL-60 group and to 2.14 ± 0.12-fold (*p* < 0.001) in the HNECs + differentiated HL-60 + IL-5 group. Consistently, wound closure at 48 h was significantly greater in the HNECs + differentiated HL-60 group (74.8 ± 5.2%, *p* = 0.003) and further enhanced in the IL-5–treated group (91.9 ± 2.7%, *p* < 0.001) compared with untreated controls (51.3 ± 0.4%).

These findings demonstrate that eosinophil-like differentiated HL-60 cells induce EMT in HNECs via paracrine mechanisms, with IL-5 amplifying this effect.

### 3.4. Knockdown of IL-5Rα/JAK2/STAT5 Signaling in IL-5-Activated Differentiated HL-60 Cells Attenuates EMT Induction in HNECs

Differentiated HL-60 cells were transfected with si-Control or siRNAs targeting IL-5RA, JAK2, or STAT5, followed by IL-5 stimulation. Western blotting confirmed efficient knockdown of each target and preservation of the sequential IL-5Rα/JAK2/STAT5 signaling cascade. Furthermore, ELISA showed that silencing each component significantly reduced the secretion of eosinophil mediators, including ECP, EPO, IL-13, and TGF-β1 (Supplementary Figure S2).

**Figure 5:**
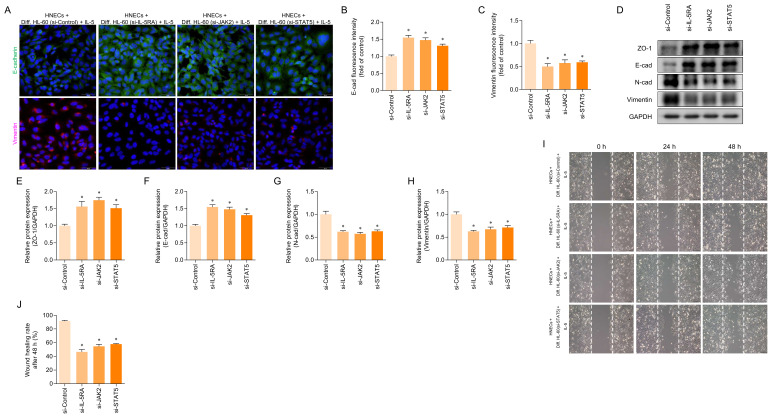
**IL-5Rα/JAK2/STAT5 signaling inhibition in eosinophil-like HL-60 cells attenuates EMT and migration in HNECs.** (**A**–**J**) HNECs were co-cultured with IL-5-activated differentiated HL-60 cells transfected with si-Control or siRNAs targeting IL-5RA, JAK2, or STAT5. (**A**) Representative immunofluorescence images of HNECs showing E-cadherin (green) and vimentin (red) expression with DAPI-stained nuclei (blue). Magnification: 400×; scale bar = 100 µm. (**B**,**C**) Quantification of relative fluorescence intensity for E-cadherin (**B**) and vimentin (**C**), expressed as fold change relative to control (HNECs co-cultured with IL-5-activated differentiated HL-60 cells transfected with si-Control). (**D**) Representative Western blot images showing EMT markers (ZO-1, E-cadherin, N-cadherin, and vimentin) in HNECs, with GAPDH as the loading control. (**E**–**H**) Densitometric quantification of ZO-1 (**E**), E-cadherin (**F**), N-cadherin (**G**), and vimentin (**H**) protein expression normalized to GAPDH. (**I**) Representative wound-healing assay images of HNECs at 0, 24, and 48 h. (**J**) Quantification of HNEC migratory capacity expressed as wound-healing rate (%) at 48 h. Data in bar graphs (**B**,**C**,**E**–**H**,**J**) are presented as the mean ± SEM from three independent biological replicates, each performed as a separate siRNA transfection and co-culture experiment. Statistical comparisons were performed using a one-way ANOVA with Tukey’s post hoc test. * *p* < 0.05 was considered statistically significant compared with the control (HNECs co-cultured with IL-5–activated differentiated HL-60 cells transfected with si-Control). Abbreviations: ANOVA, analysis of variance; DAPI, 4′,6-diamidino-2-phenylindole; Diff. HL-60, butyric acid-differentiated HL-60 cells; E-cad, E-cadherin; EMT, epithelial–mesenchymal transition; GAPDH, glyceraldehyde-3-phosphate dehydrogenase; HNECs, human nasal epithelial cells; IL-5, interleukin-5; IL-5Rα, interleukin-5 receptor α; JAK2, Janus kinase 2; N-cad, N-cadherin; SEM, standard error of the mean; si-Control, non-targeting scramble small interfering RNA; siRNA, small interfering RNA; STAT5, signal transducer and activator of transcription 5; ZO-1, zonula occludens-1.

To assess the impact on EMT, we performed immunofluorescence and Western blotting analyses of HNECs in a non-contact Transwell co-culture system. Immunofluorescence demonstrated that knockdown of IL-5RA, JAK2, or STAT5 restored E-cadherin expression to 1.55 ± 0.07-fold (*p* < 0.001), 1.48 ± 0.06-fold (*p* = 0.001), and 1.31 ± 0.05-fold (*p* = 0.02), respectively, and decreased vimentin expression to 0.51 ± 0.06-fold (*p* = 0.003), 0.58 ± 0.07-fold (*p* = 0.007), and 0.59 ± 0.03-fold (*p* = 0.008), respectively (Figure 5A–C), compared with si-Control.

Western blotting analysis yielded consistent results. ZO-1 expression increased to 1.56 ± 0.15-fold (*p* = 0.02), 1.75 ± 0.09-fold (*p* = 0.004), and 1.51 ± 0.10-fold (*p* = 0.03) following IL-5RA, JAK2, and STAT5 knockdown, respectively. Similarly, E-cadherin expression increased to 1.55 ± 0.07-fold (*p* < 0.001), 1.48 ± 0.06-fold (*p* = 0.001), and 1.31 ± 0.05-fold (*p* = 0.02) in the IL-5RA-, JAK2-, and STAT5-silenced groups, respectively. In contrast, N-cadherin expression decreased to 0.62 ± 0.02-fold (*p* = 0.02), 0.57 ± 0.03-fold (*p* = 0.01), and 0.63 ± 0.03-fold (*p* = 0.03) and vimentin expression to 0.63 ± 0.02-fold (*p* = 0.001), 0.67 ± 0.05-fold (*p* = 0.002), and 0.71 ± 0.04-fold (*p* = 0.005) in the IL-5RA-, JAK2-, and STAT5-knockdown groups, respectively (Figure 5D–H).

In line with these findings, the enhanced wound closure observed with IL-5-activated eosinophil-like cells was significantly attenuated by IL-5RA, JAK2, or STAT5 knockdown, decreasing to 46.6 ± 3.5%, 54.6 ± 2.9%, and 57.8 ± 0.9%, respectively (each *p* < 0.001), compared with 91.6 ± 0.9% in the si-Control group (Figure 5I,J).

These results demonstrate that inhibition of IL-5Rα/JAK2/STAT5 signaling in eosinophil-like cells attenuates their paracrine induction of EMT in HNECs, underscoring its role in eosinophil-mediated epithelial remodeling.

## 4. Discussion

In this study, ECRSwNP tissues were characterized by marked eosinophilic infiltration, a type 2-dominant inflammatory profile, increased IL-5Rα/JAK2/STAT5 pathway activation, and altered EMT marker expression. Although these tissue findings indicate an association between IL-5-related signaling and epithelial remodeling in ECRSwNP, they do not establish a causal relationship. To further investigate this mechanistic link, we used a non-contact co-culture system with targeted siRNA knockdown and showed that IL-5-activated eosinophils induce EMT-associated changes in HNECs via paracrine signaling regulated by the IL-5Rα/JAK2/STAT5 pathway.

Previous studies have shown that type 2 cytokines can induce EMT in airway epithelial cells. Wu et al. showed that IL-13 and IL-4 induce EMT in mouse bronchial epithelial cells, while Burgess et al. found that IL-13 induces EMT-related characteristics in human bronchial epithelial cells [19,20]. More recently, Chen et al. reported that IL-5Rα is upregulated in lung epithelial cells in pulmonary fibrosis, and that this upregulation promotes EMT through JAK2/STAT3 pathway activation [21]. With regard to HNECs, Chen et al. identified that IL-4 directly induces EMT in HNECs through the STAT6/interferon regulatory factor 4 (IRF4) pathway in ECRSwNP. Moreover, using a co-culture system, they showed that IL-4/STAT6/IRF4-driven M2 macrophages indirectly promote EMT in epithelial cells [22]. However, few studies have specifically examined the role of type 2 cytokines in EMT in HNECs, and although ECRSwNP is marked by eosinophilic inflammation, the contribution of IL-5 to EMT in HNECs remains largely unexplored. In this context, our findings offer insights into the role of IL-5-related pathways in epithelial remodeling in ECRSwNP. Nevertheless, the role of IL-5-mediated eosinophil–epithelial interaction should be considered within the broader context of type 2 inflammatory remodeling in CRSwNP. Established studies have highlighted IL-4/IL-13-driven fibrin dysregulation as a central mechanism of edematous polyp formation, in which reduced tissue plasminogen activator expression limits plasmin generation and promotes fibrin accumulation [23,24]. IL-4/IL-13 signaling may also contribute to fibrin matrix stabilization through alternatively activated macrophages and Factor XIII-A expression [25,26]. In this context, our findings do not suggest that IL-5-induced EMT is the principal driver of nasal polyp formation; rather, they indicate that IL-5-activated eosinophils may contribute to epithelial remodeling as one component of the broader type 2 inflammatory network.

Among IL-5-related signaling pathways, the IL-5Rα/JAK2/STAT5 axis is well recognized for regulating eosinophil differentiation, proliferation, and maturation as well as recruitment to inflamed tissues [27,28,29,30]. In our study, knockdown of IL-5Rα/JAK2/STAT5 significantly reduced eosinophil-derived mediator levels, including ECP, EPO, IL-13, and TGF-β1 (Supplementary Figure S2), supporting its functional role in eosinophil activation. TGF-β1 is a well-established EMT inducer in HNECs, and EPO has been reported to induce EMT in bronchial epithelial cells [11,31,32]. In this context, eosinophil-derived mediators such as TGF-β1 and EPO, released downstream of IL-5Rα/JAK2/STAT5 signaling, may promote EMT in HNECs via paracrine mechanisms. However, as this study focused on intracellular signaling in eosinophils, we did not assess the specific contributions of individual mediators, and further studies are needed to define their roles in EMT induction of HNECs.

This study has some limitations. First, it relied on an in vitro Transwell co-culture system. Although suitable for isolating paracrine effects, it does not fully reflect the complexity of in vivo tissue interactions among multiple cell types or permit direct eosinophil–epithelial contact. Given that a previous study has demonstrated EMT induction through direct contact between eosinophils and human bronchial epithelial cells, further in vivo investigations are warranted to clarify the relative contributions of paracrine versus contact-dependent pathways [33]. In addition, although IL-5Rα/JAK2/STAT5 pathway inhibition reduced several eosinophil-derived mediators within this paracrine co-culture setting, including TGF-β1, IL-13, ECP, and EPO, their individual or combined contributions to EMT induction were not determined. Moreover, the potential involvement of other eosinophil-derived soluble factors could not be excluded. Future studies using mediator-specific neutralization or receptor blockade are needed to clarify the causal mediators responsible for eosinophil-induced EMT. Second, we used butyric acid-differentiated HL-60 cells as a surrogate model for eosinophils. This model was selected because primary human eosinophils have a short ex vivo lifespan, show substantial donor-to-donor variability, and are difficult to obtain in sufficient numbers for repeated mechanistic experiments such as cytokine stimulation, co-culture, and siRNA-mediated knockdown [34,35,36]. HL-60-derived eosinophil-like models, including butyrate-differentiated HL-60 sublines, have been employed in previous studies as experimental surrogates for eosinophils [37,38,39]. In our study, these cells displayed eosinophil-like characteristics, including increased IL5RA and CCR3 expression and enhanced secretion of eosinophil-associated mediators after IL-5 stimulation. Nevertheless, HL-60-derived eosinophil-like cells cannot fully recapitulate the biological and functional properties of primary human eosinophils, particularly tissue-infiltrating eosinophils in ECRSwNP. Therefore, further validation using primary peripheral blood or tissue eosinophils from patients with ECRSwNP is needed to confirm the physiological relevance of the IL-5Rα/JAK2/STAT5-dependent responses observed in this study. Third, although serum-free medium was used to minimize cell proliferation during the wound-healing assay, mitomycin C was not used to completely block proliferative activity. Therefore, residual cell proliferation may have partially contributed to wound closure. Accordingly, these findings should be interpreted as changes in wound-closure capacity associated with migratory activity, rather than as a direct measure of migration alone. Finally, IL-5Rα activation engages additional signaling pathways, and JAK2 can also trigger other downstream cascades, including Ras/Raf-1 signaling [15], that may contribute to EMT independently or via crosstalk with JAK2/STAT5. However, we did not examine their interactions or potential compensatory activation following JAK2/STAT5 inhibition in this study.

Despite these limitations, our study suggests that IL-5-activated eosinophils may facilitate EMT-associated changes in HNECs through the release of eosinophil-derived mediators, potentially regulated by IL-5Rα/JAK2/STAT5 signaling. Biologics targeting type 2 inflammatory pathways are increasingly used in severe or refractory CRSwNP, including IL-5-targeted therapy such as mepolizumab, while IL-4/IL-13 pathway blockade, particularly with dupilumab, has shown robust clinical efficacy, and upstream epithelial alarmin-targeted approaches such as thymic stromal lymphopoietin (TSLP) inhibition are also emerging [40,41,42]. In this therapeutic context, our findings suggest that targeting the IL-5–IL-5Rα signaling axis may contribute to clinical improvement not only by reducing eosinophil burden but also by attenuating eosinophil-driven epithelial remodeling. Thus, the IL-5Rα/JAK2/STAT5 axis may represent a potential therapeutic target for modulating tissue remodeling and nasal polyp formation in ECRSwNP, a representative type 2 CRS endotype.

## 5. Conclusions

This study suggests that IL-5-induced activation of IL-5Rα/JAK2/STAT5 signaling in eosinophils promotes soluble mediator release, thereby indirectly inducing EMT-related changes in HNECs through a paracrine mechanism and contributing to tissue remodeling in ECRSwNP. The attenuation of this eosinophil-mediated effect by targeted siRNA knockdown supports further investigation of this axis as a potential therapeutic target for tissue remodeling and polyp formation in type 2 CRS, although confirmation using primary eosinophils and in vivo models is required.

## Data Availability

The original contributions presented in this study are included in the article and Supplementary Material. Further inquiries can be directed to the corresponding author.

## Supplementary Materials

The following supporting information can be downloaded at https://www.mdpi.com/article/10.3390/medicina62071360/s1, Figure S1: Differentiation and activation of HL-60 cells into eosinophil-like cells and activation of IL-5Rα/JAK2/STAT5 signaling pathway. Figure S2: siRNA knockdown efficiency and its effect on IL-5Rα/JAK2/STAT5 signaling and effector molecule release. Table S1: Primer sequences used for qRT-PCR. Table S2: Baseline demographic and clinical characteristics of the study groups.
