# Supplementary material for: Eosinophil IL-5Rα/JAK2/STAT5 Signaling Contributes to Epithelial–Mesenchymal Transition in Eosinophilic Chronic Rhinosinusitis with Nasal Polyps
Source: Medicina (Kaunas). 2026 Jul 15;62(7):1360. doi: 10.3390/medicina62071360 (PMC13413733; doi:10.3390/medicina62071360)
Supplement: Supplementary file 1 [file medicina-62-01360-s001.zip › Supplementary Figure legends.pdf]

Supplementary Figure S1. Differentiation and activation of HL-60 cells into eosinophil-like cells and activation of IL-5Rα/JAK2/STAT5 signaling pathway.

(A, B) Relative mRNA expression of IL-5RA (A) and CCR3 (B) in undifferentiated HL-60 cells, butyric acid-differentiated HL-60 cells, and differentiated cells subsequently stimulated with IL-5. Expression was determined using qRT-PCR and normalized to GAPDH. (C–F) Release of eosinophil effector molecules and cytokines from HL-60 cells after differentiation and IL-5 stimulation. Culture supernatants were analyzed for ECP (C), EPO (D), IL-13 (E), and TGF-β1 (F) using ELISA. Results are expressed in pg/mL. (G) Representative western blotting images showing IL-5R**α** protein expression and phosphorylation of downstream signaling molecules (p-JAK2/JAK2, p-STAT5/STAT5) across the three experimental groups. GAPDH served as the loading control. (H–J) Densitometric quantification of IL-5R**α** protein expression normalized to GAPDH (H) and phosphorylation levels of JAK2 (I) and STAT5 (J) expressed as phosphorylated/total protein ratios. Data are presented as the mean ± SEM from three independent biological replicates. Statistical analysis was performed using one-way ANOVA followed by Tukey's post hoc test. *$P < 0.05$ was considered statistically significant for pairwise comparisons; ns indicates statistically not significant.

Abbreviations: ANOVA, analysis of variance; CCR3, C-C chemokine receptor type 3; Diff. HL-60, butyric acid-differentiated HL-60 cells; ECP, eosinophil cationic protein; ELISA, enzyme-linked immunosorbent assay; EPO, eosinophil peroxidase; GAPDH, glyceraldehyde-3-phosphate dehydrogenase; IL-5Rα, interleukin-5 receptor α; IL-13, interleukin-13; JAK2, Janus kinase 2; p-JAK2, phosphorylated Janus kinase 2; p-STAT5, phosphorylated signal transducer and activator of transcription 5; qRT-PCR, quantitative real-time polymerase chain reaction; SEM, standard error of the mean; STAT5, signal transducer and activator of transcription 5; TGF-β1, transforming growth factor-β1; Undiff. HL-60, undifferentiated HL-60 cells.

Supplementary Figure S2. siRNA knockdown efficiency and its effect on IL-5Rα/JAK2/STAT5 signaling and effector molecule release.

(A–F) Differentiated HL-60 cells were transfected with si-Control or siRNAs targeting IL-5RA, JAK2, or STAT5 and then stimulated with IL-5. (A) Representative western blotting images showing IL-5Rα, p-JAK2, JAK2, p-STAT5, and STAT5 protein expression, with GAPDH as the loading control. (B–F) Densitometric quantification of IL-5Rα (B), p-JAK2 (C), total JAK2 (D), p-STAT5 (E), and total STAT5 (F) protein expression normalized to

GAPDH. (G–J) Quantification of eosinophil effector molecule and cytokine release from siRNA-transfected, IL-5-stimulated differentiated HL-60 cells. Culture supernatants were analyzed for ECP (G), EPO (H), IL-13 (I), and TGF-β1 (J) using ELISA. Results are expressed in pg/mL. Data are presented as the mean ± SEM from three independent biological replicates, each performed as a separate siRNA transfection experiment. Statistical analysis was performed using one-way ANOVA followed by Tukey's post hoc test for multiple comparisons. *$P < 0.05$ was considered statistically significant compared with the control (IL-5–activated differentiated HL-60 cells transfected with si-Control).

Abbreviations: ANOVA, analysis of variance; ECP, eosinophil cationic protein; ELISA, enzyme-linked immunosorbent assay; EPO, eosinophil peroxidase; IL-5, interleukin-5; IL-5Rα, interleukin-5 receptor α; JAK2, Janus kinase 2; p-JAK2, phosphorylated Janus kinase 2; p-STAT5, phosphorylated signal transducer and activator of transcription 5; TGF-β1, transforming growth factor-β1; GAPDH, glyceraldehyde-3-phosphate dehydrogenase; SEM, standard error of the mean; si-Control, non-targeting scramble small interfering RNA; siRNA, small interfering RNA; STAT5, signal transducer and activator of transcription 5; TGF-β1, transforming growth factor-β1.
