# Supplementary material for: Eosinophil IL-5Rα/JAK2/STAT5 Signaling Contributes to Epithelial–Mesenchymal Transition in Eosinophilic Chronic Rhinosinusitis with Nasal Polyps
Source: Medicina (Kaunas). 2026 Jul 15;62(7):1360. doi: 10.3390/medicina62071360 (PMC13413733; doi:10.3390/medicina62071360)
Supplement: Supplementary file 1 [file medicina-62-01360-s001.zip › Supplementary Table S1.pdf]

Supplementary Table S1. Primer sequences used for qRT-PCR.

Target gene	Accession No.	Direction	Sequence (5′–3′)	Product size (bp)
**IL-4**	NM_000589	Forward	CGAGTTGACCGTAACAGACAT	175
		Reverse	CGTCTTTAGCCTTTCCAAGAAG	
**IL-5**	NM_000879	Forward	AGCTGCCTACGTGTATGCCA	142
		Reverse	GCAGTGCCAAGGTCTCTTTCA	
**IL-13**	NM_002188	Forward	GCAATGGCAGCATGGTATGG	128
		Reverse	AAGGAATTTTACCCCTCCCTAACC	
**IFN-γ**	NM_000619	Forward	ATTCGGTAACTGACTTGAATGTCC	155
		Reverse	CTCTTCGACCTCGAAACAGC	
**IL-17A**	NM_002190	Forward	TCAACCCGATTGTCCACCAT	138
		Reverse	GAGTTTAGTCCGAAATGAGGCTG	
**IL-22**	NM_020525	Forward	CATGCAGGAGGTGGTACCTT	162
		Reverse	CAGACGCAAGCATTTCTCAG	
**IL-5RA**	NM_000590	Forward	CCTTCTCTTCCAGCTTTGCAC	110
		Reverse	AGTCCTTGACGCACACAACA	
**CCR3**	NM_001837	Forward	GCCATTTCGGACCTGCTCTT	145
		Reverse	TCCGCTCACAGTCATTTCCA	
**GAPDH**	NM_002046	Forward	AACTTTGGCATTGTGGAAGG	223
		Reverse	ACACATTGGGGGTAGGAACA	

Note. Primer sequences are presented in the 5′ to 3′ direction. **GAPDH** was used as the internal reference gene for normalization of target gene expression.

Abbreviations. bp, base pair; CCR3, C-C chemokine receptor type 3; GAPDH, glyceraldehyde-3-phosphate dehydrogenase; IFN-γ, interferon gamma; IL, interleukin; qRT-PCR, quantitative real-time polymerase chain reaction.
