# Supplementary material for: Eosinophil IL-5Rα/JAK2/STAT5 Signaling Contributes to Epithelial–Mesenchymal Transition in Eosinophilic Chronic Rhinosinusitis with Nasal Polyps
Source: Medicina (Kaunas). 2026 Jul 15;62(7):1360. doi: 10.3390/medicina62071360 (PMC13413733; doi:10.3390/medicina62071360)
Supplement: Supplementary file 1 [file medicina-62-01360-s001.zip › Supplementary Table S2.pdf]

Supplementary Table S2. Baseline demographic and clinical characteristics of the study groups.

	Control (n=12)	Non-ECRSwNP (n=12)	ECRSwNP (n=12)	*P*-value
**Age, years**	43.5 (37.5-65.3)	59.0 (39.5-64.8)	49.0 (36.5-59.8)	0.549
**Sex, male/female**	8/4	8/4	9/3	1.000
**Asthma, n (%)**	0 (0.0)	0 (0.0)	2 (16.7)	0.314
**Allergic rhinitis, n (%)**	3 (25.0)	2 (16.7)	3 (25.0)	1.000
**Blood eosinophils, %**	1.3 (1.0-1.9)	1.4 (0.9-2.0)	7.8 (5.0-10.5)	<0.001*
**Blood eosinophil count, cells/μL**	106.1 (51.3-118.7)	77.3 (50.3-136.0)	498.5 (304.8-765.0)	<0.001*
**Total IgE, IU/mL**	20.3 (4.5-51.9)	16.2 (7.8-132.5)	133.5 (89.5-263.3)	0.016*
**Lund-Mackay score**	2.0 (1.0-4.8)	11.5 (9.0-13.0)	13.0 (10.0-15.5)	<0.001*

Note. Data are presented as median (interquartile range) or number (%). *P* values indicate overall comparisons among the three groups and were calculated using available data for each variable. Continuous variables were compared using the Kruskal–Wallis test, and categorical variables were compared using Fisher's exact test. Allergic rhinitis was defined based on MAST positivity. Total IgE values were missing for 1 subject in the control group, 3 subjects in the non-ECRSwNP group, and 6 subjects in the ECRSwNP group. **P* < 0.05 was considered statistically significant.

Abbreviations. ECRSwNP, Eosinophilic chronic rhinosinusitis with nasal polyps; MAST, Multiple allergen simultaneous test
