# Supplementary figures and images for: Eosinophil IL-5Rα/JAK2/STAT5 Signaling Contributes to Epithelial–Mesenchymal Transition in Eosinophilic Chronic Rhinosinusitis with Nasal Polyps
Source: Medicina (Kaunas). 2026 Jul 15;62(7):1360. doi: 10.3390/medicina62071360 (PMC13413733; doi:10.3390/medicina62071360)

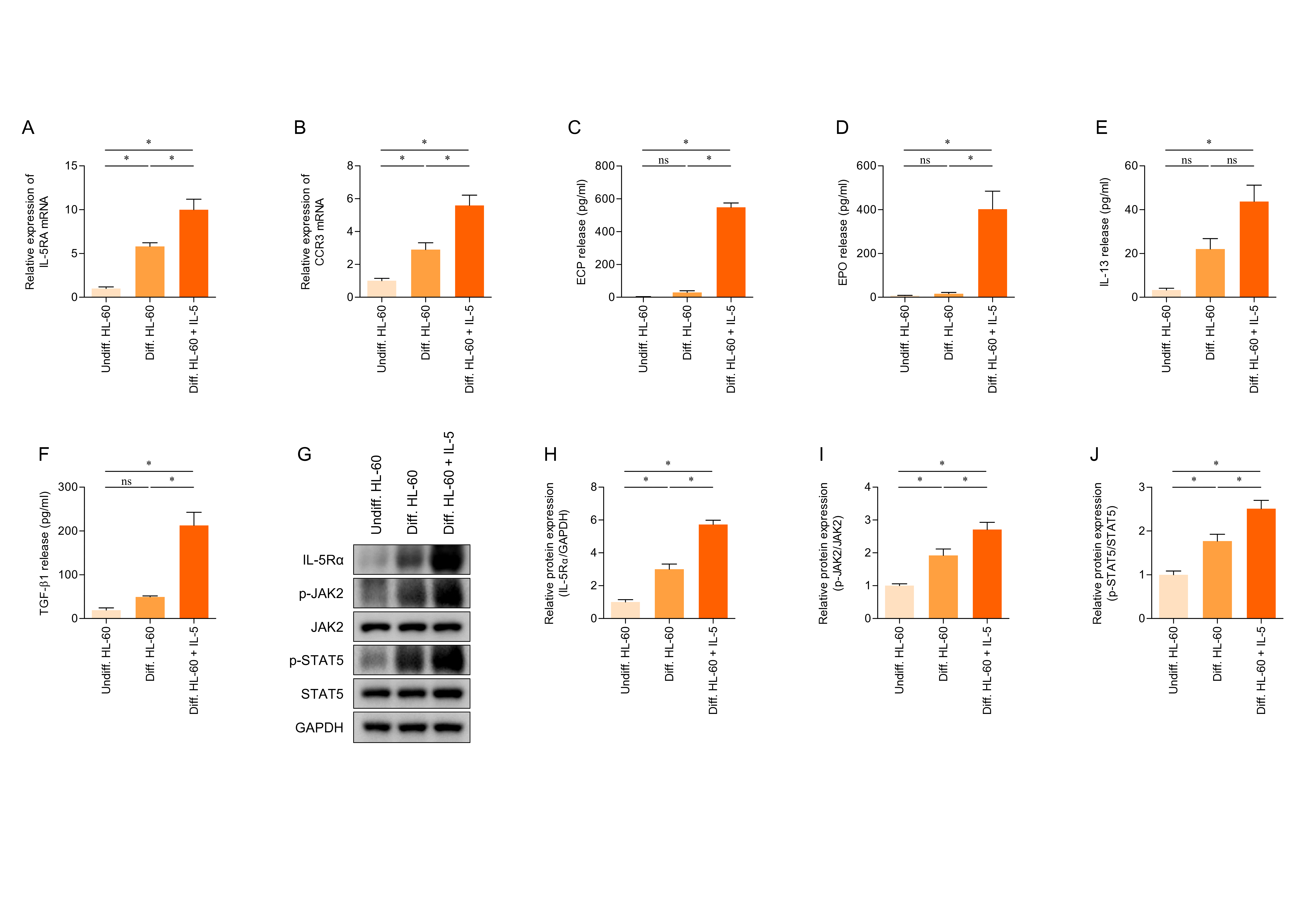

Supplement: Supplementary file 1 [file medicina-62-01360-s001.zip › Figure S1.tiff]

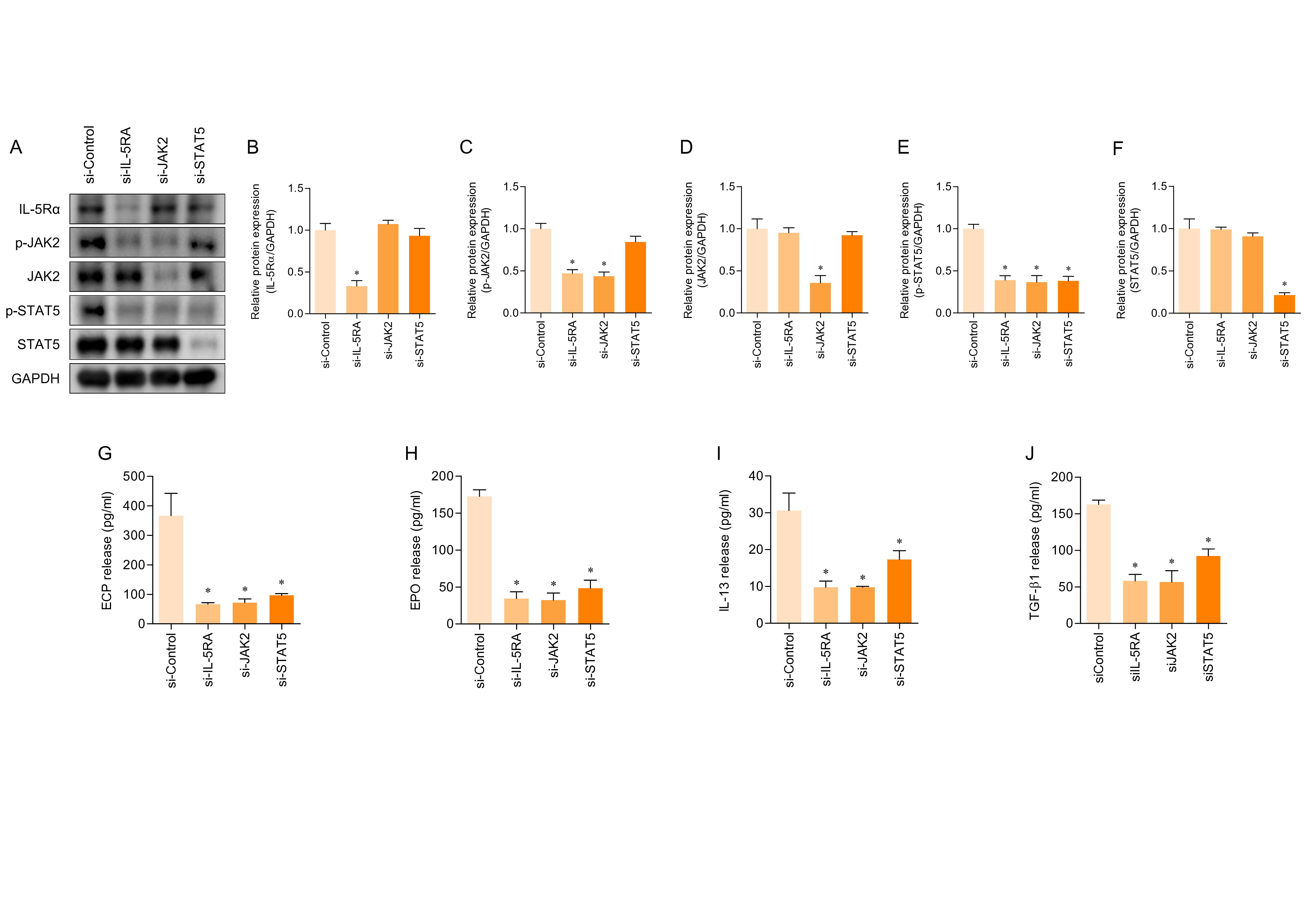

Supplement: Supplementary file 1 [file medicina-62-01360-s001.zip › Figure S2.tiff]
